# Supplementary material for: Is Gender an Antecedent to Workplace Stressors? A Systematic Review and an Empirical Study Using a Person-Centred Approach
Source: Int J Environ Res Public Health. 2023 Apr 17;20(8):5541. doi: 10.3390/ijerph20085541 (PMC10139098; doi:10.3390/ijerph20085541)
Supplement: Supplementary file 1 [file ijerph-20-05541-s001.zip › ijerph-2273503-supplementary.pdf]

***Table S1*. Fit Indices of the Alternative Factor Models for the MS-IT Tool for Each Gender Group.**

	MLRχ^2	*df*	RMSEA (90% C.I.)	CFI	SRMR	AIC	ECVI
**Males (n=5,716)**							
CFA Model	6621,767	539	,044 (044-.045)	.875	.060	48.358.807	26.277
ESEM Model	2574,514	371	.032 (.030-.034)	.955	.016	465,342.456	3.470
BIFACTOR Model	5212,143	525	.040 (.039-.041)	.900	.046	478,170.264	18.615
**Females (n=5,573)**							
CFA Model	7034,538	539	.047 (.046-.047)	.867	.061	467,661.368	27.915
ESEM Model	2977,207	371	.036 (.034-.037)	.946	.019	459,927.257	4.012
BIFACTOR Model	6246,469	525	.044 (.034-.037)	.882	.053	466,222.251	22.309

Note. MLR = Robust Maximum Likelihood; df = degrees of freedom; RMSEA = Root Mean Square Error of Approximation; CFI = Comparative fit Index; SRMR = Standardized Root Mean Square Residual; AIC = Akaike Information Criterion; ECVI = Expected Cross-Validation Index.

***Table S2*. Gender Measurement Invariance Models of the Final Oblique ESEM 6-Factor Model.**

	MLRχ^2	*df*	RMSEA (90% C.I.)	CFI	SRMR	Model Comparison	ΔCFI
**CONFIGURAL**	7,029	800	.037 (.036 - .038)	.935	.021	-	
**METRIC**	7,172.407	974	.034 (.033 - .034)	.936	.025	Metric vs. Configural	-.001
**SCALAR**	7,486.984	1003	.034 (.033 - .034)	.934	.027	Scalar vs. Metric	.002
**STRICT**	7,383.691	1038	.033 (.033 - .034)	.935	.030	Strict vs. Scalar	-.001

Note. MLR = Robust Maximum Likelihood; df = degrees of freedom; RMSEA = Root Mean Square Error of Approximation; CFI = Comparative fit Index; SRMR = Standardized Root Mean Square Residual.

***Table S3*. Factor Loadings on the Strict Invariance 6-Factor ESEM Model.**

	ESEM Factor 1		ESEM Factor 2		ESEM Factor 3		ESEM Factor 4		ESEM Factor 5		ESEM Factor 6	
	Role Clarity		Control		Demands		Poor Relationships		Peer Support		Management Support	
	Males	Females	Males	Females	Males	Females	Males	Females	Males	Females	Males	Females
**Item 1**	**.62**	**.61**	.04	.04	.03	.03	-.09	-.09	.03	.03	.07	.07
**Item 4**	**.55**	**.54**	.15	.15	-.07	-.08	.00	.00	.01	.01	-.14	-.15
**Item 11**	**.73**	**.73**	.04	.04	-.02	-.02	-.04	-.04	.04	.04	-.03	-.03
**Item 13**	**.70**	**.69**	-.03	-.03	.00	.00	-.04	-.05	.01	.01	.18	.18
**Item 17**	**.67**	**.67**	.02	.02	-.02	-.02	-.01	-.01	.00	.00	.15	.15
**Item 2**	-.21	-.20	**.57**	**.54**	-.14	-.15	-.03	-.03	.02	.02	-.04	-.04
**Item 10**	.11	.10	**.62**	**.60**	-.06	-.07	.03	.03	.04	.04	.02	.02

**Item 15**	.09	.09	**.78**	**.76**	.04	.04	-.04	-.04	-.05	-.06	.00	.00
**Item 19**	.00	.00	**.74**	**.72**	.00	.00	.04	.04	-.03	-.03	.01	.01
**Item 25**	.13	.13	**.55**	**.53**	.08	.09	-.05	-.05	.09	.09	.16	.17
**Item 30**	-.11	-.11	**.37**	**.35**	.00	.00	-.02	-.02	.06	.06	.14	.14
**Item 3**	-.10	-.10	-.06	-.06	**.52**	**.54**	.02	.02	-.03	-.03	-.02	-.02
**Item 6**	-.12	-.12	-.02	-.01	**.68**	**.70**	.04	.04	-.01	-.01	.02	.02
**Item 9**	.26	.24	.05	.05	**.69**	**.70**	-.02	-.02	.02	.02	-.02	-.02
**Item 12**	-.07	-.06	.06	.06	**.70**	**.72**	-.01	-.01	.00	.00	-.01	-.01
**Item 16**	.21	.20	-.24	-.23	**.40**	**.41**	.06	.06	-.04	-.04	.00	.00
**Item 18**	.06	.06	-.19	-.18	**.34**	**.36**	.17	.18	.02	.02	-.03	-.03
**Item 20**	.20	.18	.01	.01	**.66**	**.68**	-.01	-.01	-.01	-.01	-.06	-.06
**Item 22**	-.05	-.05	-.05	-.05	**.69**	**.72**	.07	.07	.00	.00	-.01	-.01
**Item 5**	-.02	-.02	-.03	-.02	.04	.04	**.76**	**.78**	-.01	-.01	.00	.00
**Item 14**	-.06	-.06	.08	.08	.07	.07	**.41**	**.43**	-.31	-.31	-.03	-.03
**Item 21**	-.02	-.02	-.03	-.03	.04	.04	**.76**	**.78**	-.01	-.01	-.05	-.05
**Item 34**	.00	.00	-.01	-.01	.02	.02	**.32**	**.33**	-.17	-.17	.00	.00
**Item 7**	.00	.00	-.01	-.01	-.07	-.07	.02	.02	**.74**	**.75**	-.01	-.01
**Item 24**	.01	.01	-.02	-.01	.03	.04	-.05	-.05	**.83**	**.83**	.01	.01
**Item 27**	.02	.02	.05	.05	.10	.10	-.34	-.35	**.50**	**.50**	.01	.01
**Item 31**	-.06	-.06	.06	.06	.07	.07	-.08	-.08	**.71**	**.71**	.06	.06
**Item 8**	.08	.08	.01	.01	-.08	-.09	.04	.05	.37	.38	**.29**	**.29**
**Item 23**	.00	.00	-.02	-.02	-.02	-.02	-.07	-.07	.02	.02	**.73**	**.73**
**Item 29**	-.03	-.03	.02	.02	.04	.04	-.10	-.11	-.01	-.01	**.79**	**.79**
**Item 33**	.03	.03	.03	.03	-.07	-.07	.02	.02	.35	.37	**.38**	**.39**
**Item 35**	-.02	-.02	-.05	-.04	.06	.07	-.03	-.03	-.03	-.03	**.88**	**.88**
**Item 26**	.08	.08	.12	.12	-.03	-.04	.02	.02	.02	.03	**.62**	**.63**
**Item 28**	.11	.10	.04	.04	-.06	-.06	.02	.02	.06	.07	**.57**	**.58**
**Item 32**	.36	.35	.04	.04	-.04	-.04	.06	.06	.11	.11	**.34**	**.36**

Note. Factor loadings on the target factor are reported in bold.

Table S4. **Principal Effects of the Two-Way Factorial MANOVA.**

Factor	Variable	gdl₁ (gdl₂=11,283)	F	*p*	partial η^2
**Profile Membership**					
	Role Clarity	2	2,778.470	< .001	.330
	Control	2	3,424.436	< .001	.378
	Demands	2	1,304.807	< .001	.188
	Poor Relationships	2	5,088.531	< .001	.474
	Peer Support	2	6,852.763	< .001	.548
	Management Support	2	10,688.022	< .001	.655
**Gender**					
	Role Clarity	1	9.906	< .01	.001
	Control	1	51.896	< .001	.005
	Demands	1	13.591	< .001	.001
	Poor Relationships	1	15.561	< .001	.001
	Peer Support	1	1.228	.268	0
	Management Support	1	13.021	< .001	.001
**Profile Membership*Gender**					
	Role Clarity	2	48.137	< .001	.008
	Control	2	6.824	.001	.001
	Demands	2	22.671	< .001	.004
	Poor Relationships	2	9.678	< .001	.002
	Peer Support	2	.646	.524	0
	Management Support	2	5.608	< .01	.001
